# Efficacy and safety of guselkumab and adalimumab for pustulotic arthro-osteitis and their impact on peripheral blood immunophenotypes

**DOI:** 10.1186/s13075-022-02934-3

**Published:** 2022-10-27

**Authors:** Masanobu Ueno, Ippei Miyagawa, Yusuke Miyazaki, Kentaro Hanami, Shunsuke Fukuyo, Satoshi Kubo, Shingo Nakayamada, Yoshiya Tanaka

**Affiliations:** 1 The First Department of Internal Medicine, School of Medicine, University of Occupational and Environmental Health, 1-1 Iseigaoka, Kitakyushu, 807-8555 Japan; 2 Department of Internal Medicine 3, Rheumatology and Immunology, Friedrich-Alexander-University Erlangen-Nürnberg (FAU) and Universitätsklinikum Erlangen, Erlangen, Germany; 3 Deutsches Zentrum für Immuntherapie (DZI), FriedrichAlexander-University Erlangen-Nürnberg (FAU) and Universitätsklinikum Erlangen, Erlangen, Germany

**Keywords:** Palmoplantar pustulosis, Pustulotic arthro-osteitis, Guselkumab, Adalimumab

## Abstract

**Objectives:**

We compared the treatment effectiveness between guselkumab and adalimumab in patients with pustulotic arthro-osteitis (PAO). In addition, we performed peripheral blood immunophenotyping to elucidate the immunological background and analyzed the impact of therapeutic drugs to verify the validity of immunological phenotypes as therapeutic targets.

**Methods:**

Patients were treated with guselkumab 100 mg (guselkumab group; *n* = 12) and adalimumab 40 mg (adalimumab group; *n* = 13). Arthritis disease activity, skin lesion activity, and patient-reported outcomes (PROs) were evaluated and compared between the two groups. The retention rate and adverse events were evaluated. Comprehensive phenotyping of peripheral immune cells was performed in both groups, and phenotypes were compared before and after treatment.

**Results:**

At 6 months, both groups showed significant improvement in arthritis disease activity and PROs. In the guselkumab group, skin symptoms significantly improved. The 6-month continuation rates were 91.7% (11/12) and 69.2% (9/13) in the guselkumab and adalimumab groups, respectively. Adverse events occurred in 2/12 and 5/13 patients in the guselkumab (16.7%) and adalimumab (38.5%) groups, respectively. Peripheral blood immunophenotyping showed that the proportion of activated T helper (Th) 1 cells was significantly lower in patients with PAO than in healthy controls and that the proportion of activated Th17 cells was significantly higher in patients with PAO, which significantly decreased after treatment with guselkumab.

**Conclusion:**

Although guselkumab and adalimumab have comparable efficacy for PAO, their impact on immunophenotypes varies.

**Supplementary Information:**

The online version contains supplementary material available at 10.1186/s13075-022-02934-3.

## Background

Palmoplantar pustulosis (PPP) is a chronic and recurrent inflammatory skin disease that is characterized by the appearance of sterile pustules after the formation of erythematous and scaly vesicles localized to the palms and soles [1, 2]. PPP is complicated by arthro-osteitis in approximately 10–45% of cases and is referred to as pustulotic arthro-osteitis (PAO) [3, 4]. PAO exhibits various joint symptoms such as sternocostoclavicular arthritis, spondylitis, sacroiliitis, and peripheral arthritis.

Serum tumor necrosis factor (TNF)-α is known to be elevated in PPP [5]. Anti-TNF inhibitors have been used as a treatment for PPP; however, studies showed controversial efficacy [6, 7], and further verification was needed. Guselkumab is a biological agent that binds to the p19 subunit protein of interleukin (IL)-23 and selectively inhibits IL-23 signaling. Recent clinical studies have reported the efficacy of guselkumab for PPP. In Japan, the National Health Insurance System has covered guselkumab since November 2018 for the treatment of PPP that responds inadequately to conventional treatments [8, 9]. Although clinical studies have reported an exploratory analysis of the efficacy of guselkumab for joint symptoms of PPP, its efficacy in real-world clinical practice has only been described in case reports and has not yet been fully verified [10, 11].

The IL-12/T helper (Th) 1 and IL-23/Th17 cell axes have been reported to play important roles in the pathogenesis of PPP and PAO. Especially in skin lesions, activation of the IL-23/Th17 cell axis has been considered to play a central role in the pathology of both diseases, and the expression of cytokines produced by Th17 cells, such as IL-17A and IL-17F, is markedly increased in skin lesions [5, 12, 13]. However, an analysis of the peripheral blood immunophenotypes in samples from patients with PAO has not been conducted, and the impact of treatment on these phenotypes is also unknown. In this study, we compared guselkumab and adalimumab for the treatment of PAO in terms of efficacy and safety. In addition, we performed peripheral blood immunophenotyping to elucidate the immunological background of PAO and analyzed the impact of therapeutic drugs to verify the validity of phenotypes as therapeutic targets.

## Patients and methods

### Patients and clinical measurements

The subjects were patients with PAO who presented with rash and joint symptoms refractory to conventional treatments and administered guselkumab 100 mg (12 patients) or adalimumab 40 mg (13 patients) at our hospital and affiliated institutions between January 2015 and September 2021. Since guselkumab was covered as the only available bDMARDs (biological disease-modifying antirheumatic drugs [DMARDs]) for refractory PAO by the National (Japanese) Health Insurance System in November 2018, guselkumab has been introduced in all cases of treatment-resistant PAO at our facility. Prior to that, adalimumab was used for treatment-resistant PAO but was not administered after November 2018. To minimize the difference in the levels of treatment with drugs other than guselkumab and adalimumab, the adalimumab group included patients who started treatment during a 3-year period up to November 2018 (*n* = 13), while the guselkumab group included those who started treatment during a 3-year period from November 2018 to September 2021 (*n* = 12). Patient data were retrospectively collected for up to 6 months after treatment initiation. All patients started guselkumab or adalimumab therapy after receiving standard treatment for PPP/PAO. In this study, the standard treatment was defined as the use of topical corticosteroids, topical vitamin D3, phototherapy, and immunosuppressants (methotrexate, cyclosporine, and salicylazosulfapyridine).

The primary endpoints were the achievement rate of low disease activity (LDA) in disease activity in psoriatic arthritis (DAPSA) (DAPSA-LDA: DAPSA ≤ 14) and the achievement rate of remission (REM) in DAPSA (DAPSA-REM: DAPSA ≤ 4) at 6 months. The secondary endpoint was the Palmoplantar Pustulosis Area Severity Index (PPPASI) response rate (PPPASI-50, PPPASI-75, PPPASI-90) at 1, 3, and 6 months. Other secondary endpoints were arthritis disease activity (swollen joint counts [SJ66], tender joint counts [TJ68], Patient Global Assessment [PGA], Pain Visual Analog Scale [Pain VAS], and DAPSA), skin lesion activity (PPPASI), and patient-reported outcomes (PROs: Health Assessment Questionnaire [HAQ] and European Quality of Life score with five dimensions [EQ-5D]) at 1, 3, and 6 months. In addition, the 6-month treatment continuation rate and the presence or absence of new adverse events after the introduction of guselkumab or adalimumab were evaluated in both groups. The severity of adverse events was classified according to the National Cancer Institute Common Terminology Criteria for Adverse Events (CTCAE) version 5.0.

### Flow cytometric analysis

Immunophenotyping analysis was conducted using multicolor flow cytometry. After obtaining informed consent, blood was withdrawn. All collected samples were immediately analyzed by flow cytometry. In patients with PPP who were treated with bDMARDs, the blood samples were taken at baseline (guselkumab: *n* = 11, adalimumab: *n* = 11) and at month 6 of treatment (guselkumab: *n* = 6, adalimumab: *n* = 8). After staining with the indicated antibodies (Supplementary Table S1), cells were analyzed by multicolor flow cytometry (FACS Lyric; Becton Dickinson). Similarly, 30 healthy controls (HCs) and 34 patients with psoriatic arthritis (PsA) who were matched for age and sex were evaluated. The cells were collected and analyzed with FlowJo software (Tree Stare). The phenotype of immune cell subsets was defined based on the Human Immunology Project protocol of comprehensive 8-color flow cytometric analysis proposed by the National Institutes of Health (NIH) and the Federation of Clinical Immunology Societies (FOCIS) [14]. For instance, activated Th1 cells were defined as CD3^+^CD4^+^CXCR3^+^CCR6^−^CD38^+^ HLA-DR^+^ cells, and activated Th17 cells were defined as CD3^+^CD4^+^CXCR3^+^CCR6^−^CD38^+^HLA-DR^+^cells. Details of the flow cytometry antibody panels and the gating strategy are described in Supplementary Table S1 and Supplementary Fig. S1. In addition, the following values were calculated and presented for the data on the phenotypes: proportion of CD4^+^ T cell subsets and activated CD4^+^ T cells to CD3^+^ and CD4^+^ T cells (%) (Fig. S1-A, C); proportion of CD8^+^ T cells and activated CD8^+^ T cells to CD3^+^ and CD8^+^ T cells (%) (Fig. S1-B, C); proportion of B cells to CD3^−^ and CD19^+^ B cells (%) (Fig. S1-D); proportion of monocytes to CD3^−^, CD19^−^, CD20^−^, and CD14^+^ cells (%) (Fig. S1-E); proportion of myeloid dendritic cells (DCs) and plasmacytoid DCs to CD3^−^, CD19^−^, CD20^−^, CD14^−^, and human leukocyte antigen-DR^+^ cells (%) (Fig. S1-F); and proportion of CD16^+^ and CD16^−^ natural killer cells to CD3^−^, CD19^−^, CD20^−^, CD14^−^, and CD56^+^ cells (%) (Fig. S1-F).

### Serum cytokine measurement

Serum levels of cytokines (TNF-α, IL-17A) were measured in patients by electrochemiluminescence before (guselkumab; *n*=12, adalimumab; *n*=11) and 6 months after the treatment with guselkumab (*n*=5) or adalimumab (*n*=7). Serum samples were isolated immediately after taking blood from patients and stored in a −80°C freezer; serum samples were not thawed until cytokine measurement. The MESO SCALE DISCOVERY S-PLEX Human IL-17A kit (#K15067L-1, Meso Scale Diagnostics, LLC, Rockville, MD, USA) was used to measure IL-17A (fg/mL), and the U-PLEX Biomarker Group 1 (#K151C3S-1, Meso Scale Diagnostics, LLC, Rockville, MD, USA) was used to measure TNF-α (pg/mL).

### Statistical analysis

The data are expressed as median (IQR, interquartile range) or number (%). For statistical analysis, data from cases in which guselkumab or adalimumab was discontinued or relapsed were complemented using the last observation carried forward method. Differences between groups were compared using Fisher’s exact test or the Wilcoxon rank sum test. The Wilcoxon signed-rank test was used to detect statistically significant differences between each group’s baseline data and those measured at months 1, 3, and 6. Differences between the groups (the guselkumab group vs the adalimumab group) were compared using the Wilcoxon sum rank test. Dunn’s test was used to compare the results of flow cytometry analysis at baseline between HCs, patients with PsA, and patients with PAO. Spearman’s rank correlation coefficients were calculated to evaluate the correlation of changes in the results of the flow cytometric analysis before and after treatment with changes in PPPASI/DAPSA.

All reported *p* values were two-sided and were not adjusted for multiple testing. The level of significance was set at *p*<0.05. All analyses were conducted using JMP Pro version 15 (SAS Institute Inc., Cary, NC) and GraphPad Prism 9 (GraphPad Software, San Diego, CA).

## Result

### Comparison of treatment response between guselkumab and adalimumab

The patient characteristics are shown in Table 1. At baseline, no statistically significant differences between the two groups were observed in terms of age, sex, disease duration, smoking history, skin symptoms, joint symptoms, joint lesion sites, PROs, or inflammatory responses. Additionally, all patients had peripheral arthritis. There were also no statistically significant differences in the proportion of patients with lesions in the sternoclavicular joint, sacroiliac joint, or spine.

**Table 1:** Baseline characteristics of the guselkumab group (*n* = 12) and the adalimumab group (*n* = 13)

	Guselkumab group (*N*=12)	Adalimumab group (*N*=13)	*P* value
Age	53.5 (46.0, 61.8)	55.0 (48.5, 58.0)	1.0000
Male/female	1/11	3/10	0.5930
Disease duration (months)	29.0 (6.3, 73.8)	18.0 (6.5, 324)	0.4459
Age at diagnosis	50.5 (41.5, 56.8)	45.0 (31.5, 55.5)	0.2530
Peripheral joint, *n* (%)	12 (100)	13 (100)	
Sternoclavicular joint, *n* (%)	7/12 (58.3)	10/13 (76.9)	0.4110
Sacroiliac joint, *n* (%)	7/12 (58.3)	7/13 (53.9)	1.0000
Spine, *n* (%)	2/12 (16.7)	3/13 (23.1)	1.0000
Concomitant CS, *n* (%)	0 (0)	2 (16.7)	0.4857
History of csDMARDs	MTX 5, SASP 2	MTX 11, SASP 2, CyA 1	0.0730
History of TNFi, *n* (%)	4 (33.3)	2 (15.4)	0.1602
History of IL-17i, *n* (%)	1 (8.3)	0 (0)	0.4800
Weight (kg)	54.9 (48.3, 68.2)	56.1 (51.5, 72.8)	0.5496
BMI	21.7 (19.4, 27.7)	23.0 (20.5, 26.3)	0.4792
Smoking history, *n* (%)	8 (66.7)	9 (69.2)	1.0000
PPPASI	7.2 (1.7, 14.6)	3.0 (1.4, 18.6)	0.5137
Patient pain VAS (cm)	5.3 (3.0, 7.5)	4.5 (2.3, 6.6)	0.4138
Patient global VAS (cm)	5.3 (3.4, 7.5)	4.0 (3.5, 6.2)	0.3685
SJ66	1.5 (0, 3.0)	1.0 (0, 1.5)	0.3973
TJ68	4.0 (3.0, 7.3)	8.0 (3.5, 13.5)	0.1633
DAPSA	17.3 (10.3, 34.0)	19.7 (15.0, 26.8)	0.7223
HAQ	0.69 (0.28, 1.34)	0.75 (0.19, 1.50)	0.8911
EQ-5D	0.61 (0.54, 0.77)	0.61 (0.52, 0.73)	0.9738
CRP	0.13 (0.07, 0.65)	0.46 (0.05, 1.00)	0.8275
ESR	22.5 (9.0, 48.5)	19.0 (12.0, 25.0)	0.5853

Data are shown by median (quartile) or *n* (%). *p* values were determined by Fisher’s exact test or the Wilcoxon rank sum test

*CS* corticosteroid, *csDMARDs* conventional synthetic disease-modifying antirheumatic drugs, *TNFi* tumor necrosis factor inhibitor, *IL-17i* interleukin 17 inhibitor, *BMI* body mass index, *PPPASI* Palmoplantar Pustulosis Area and Severity Index, *VAS* visual analog scale, *SJ* swollen joint, *TJ* tender Joint, *DAPSA* disease activity in psoriatic arthritis, *HAQ* Health Assessment Questionnaire, *EQ-5D* EuroQOL score with 5 dimensions, *CRP* C-reactive protein, *ESR* erythrocyte sedimentation rate

*p<0.05: guselkumab group (*n*=12) vs adalimumab group (*n*=13)

**Fig. 1:**
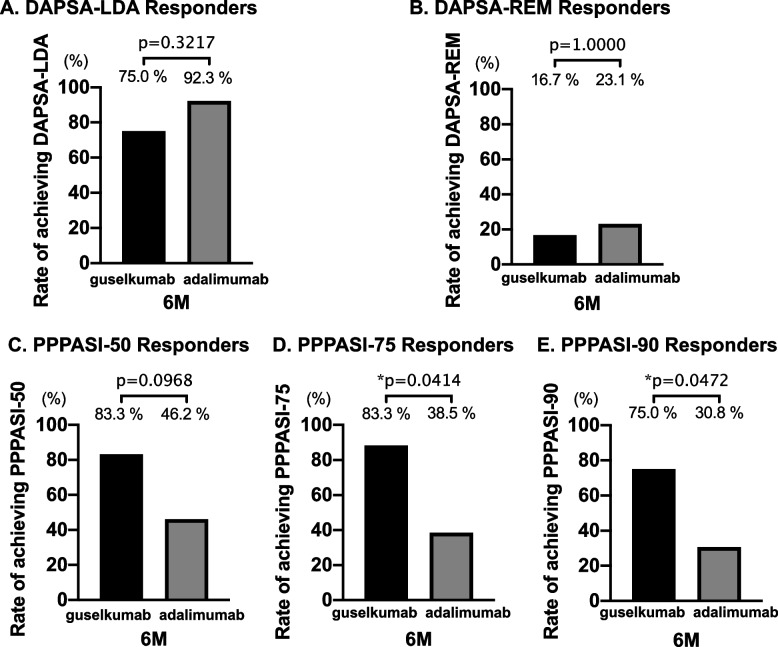
Comparison of 6 months of treatment response between the guselkumab group (*n* = 12) and adalimumab group (*n* = 13). **A** Rate of DAPSA-LDA responders (%). **B** Rate of DAPSA-REM responders. **C** Rate of PPPASI-50 responders (%). **D** Rate of PPPASI-75 responders. **E** Rate of PPPASI-90 responders (%). *p<0.05, by Fisher’s exact test. Abbreviations: PPPASI Palmoplantar Pustulosis Area Severity Index, DAPSA disease activity in psoriatic arthritis, LDA low disease activity, REM remission

Regarding the primary endpoints, there were no significant differences in DAPSA-LDA and DAPSA-REM at 6 months in both groups (Fig. 1A, B). Although the 6-month achievement rate of PPPASI-50 was not significantly different between the two groups, that of PPPASI-75 and PPPASI-90 was significantly higher in the guselkumab group (Fig. 1C–E). We show the differences in DAPSA-LDA/REM and PPPASI-50, PPPASI-75, and PPPASI-90 at 1 and 3 months between the two groups in Supplementary Fig. S2. Table 2 shows changes in the treatment responses in the guselkumab and adalimumab groups at each observation point. Regarding joint symptoms, no significant improvement in SJ66 was observed in either group during the observation period. However, TJ68, PGA results, Pain VAS scores, and DAPSA significantly improved in both groups (Table 2).

**Table 2:** Effectiveness of guselkumab and adalimumab therapy measured through eight factors over 6 months

	**Guselkumab group (*N*=12)**					
	1M	3M	6M	*p* value (1M vs 0M)	*p* value (3M vs 0M)	*p* value (6M vs 0M)
PPPASI	5.3 (1.2, 14.0)	2.3 (0, 4.7)	0 (0, 2.6)	0.3828	0.0068*	0.0039*
SJ66	0 (0, 2.3)	0 (0, 1.5)	0 (0, 1.5)	0.0625	0.1563	0.1563
TJ68	3.0 (1.3, 5.5)	2.5 (1.0, 5.5)	2.5 (1.3, 5.5)	0.0898	0.0352*	0.0410*
PGA (cm)	3.6 (3.0, 4.7)	2.8 (1.3, 4.3)	3.3 (1.2, 3.9)	0.0039*	0.0171*	0.0225*
Pain VAS (cm)	3.4 (1.8, 4.8)	2.7 (1.3, 3.8)	1.8 (1.2, 3.6)	0.0273*	0.0176*	0.0137*
DAPSA	10.1 (5.6, 19.2)	9.3 (4.4, 18.7)	8.5 (4.8, 18.1)	0.0049*	0.0210*	0.0161*
HAQ	0.38 (0.13, 1.00)	0.19 (0, 0.69)	0.25 (0.03, 0.50)	0.0039*	0.0020*	0.0059*
EQ-5D	0.73 (0.56, 0.82)	0.73 (0.64, 0.85)	0.75 (0.64, 0.85)	0.1563	0.0391*	0.0488*
CRP (mg/dL)	0.09 (0.05, 0.22)	0.14 (0.04, 0.42)	0.07 (0.03, 0.35)	0.1204	0.1403	0.0775
	**Adalimumab group (*N*=13)**					
	1M	3M	6M	*p* value (1M vs 0M)	*p* value (3M vs 0M)	*p* value (6M vs 0M)
PPPASI	2.6 (1.1, 17.5)	1.2 (0.4, 15.6)	2.4 (0.4, 14.6)	0.2500	0.6523	0.5342
SJ66	0 (0, 1.0)	0 (0, 1.5)	0 (0, 1.5)	0.0547	0.2422	0.3750
TJ68	3.0 (1.0, 8.0)	1.0 (0, 5.0)	1.0 (0, 2.0)	0.0039*	0.0010*	0.0010*
PGA	2.2 (1.0, 4.1)	2.2 (1.1, 2.9)	2.2 (1.2, 3.9)	0.0059*	0.0068*	0.0195*
Pain VAS	2.5 (9.5, 3.8)	1.2 (0.8, 2.5)	1.0 (0.8, 1.9)	0.0103*	0.0039*	0.0039*
DAPSA	8.4 (6.1, 16.1)	6.5 (2.8, 12.2)	6.7 (3.2, 8.4)	0.0005*	0.0024*	0.0015*
HAQ	0.38 (0, 0.75)	0.38 (0, 0.63)	0.25 (0, 0.75)	0.0313*	0.0156*	0.0156*
EQ-5D	0.68 (0.65, 0.75)	0.68 (0.65, 0.77)	0.68 (0.66, 0.75)	0.0234*	0.0234*	0.0391*
CRP (mg/dL)	0.07 (0.01, 0.27)	0.10 (0.04, 0.30)	0.07 (0.02, 0.78)	0.0110*	0.1078	0.2077

Data are shown by median (quartile) or *n* (%). *p* values were determined by Fisher’s exact test or the Wilcoxon signed-rank test

*PPPASI* Palmoplantar Pustulosis Area and Severity Index, *VAS* visual analog scale, *SJ* swollen joint, *TJ* tender joint, *DAPSA* disease activity in psoriatic arthritis, *HAQ* Health Assessment Questionnaire, *EQ-5D* EuroQOL score with 5 dimensions, *CRP* C-reactive protein

*p<0.05: guselkumab group (*n*=12) vs adalimumab group (*n*=13)

As for skin symptoms, the PPPASI in the guselkumab group significantly decreased at 3 months after treatment initiation and remained significantly decreased up to 6 months (Table 2). In the adalimumab group, the PPPASI did not significantly decrease during the observation period. Regarding PROs, the HAQ and EQ-5D scores significantly improved in both groups (Table 2).

### Safety profile and retention rate

Table 3 lists the reasons for discontinuation and adverse events. The 6-month retention rates, representing the primary endpoint, were noted in 11/12 and 9/13 patients in the guselkumab (91.7%) and adalimumab (69.2%) groups, respectively. One patient discontinued guselkumab because of bulbar conjunctival hyperemia. In the adalimumab group, treatment was discontinued because of psoriasiform rash in three patients and primary failure in one patient. The incidence of adverse events, including others, was noted in 2/12 patients (16.7%) in the guselkumab group and 5/13 patients (38.5%) in the adalimumab group. No adverse events of CTCAE grade ≥3 occurred in either group.

**Table 3:** Adverse events in the guselkumab group and adalimumab group

Case no.	Group	Adverse events	CTCAE grade
1	Guselkumab	None	
2	Guselkumab	3M: hepatic disfunction	1
3	Guselkumab	None	
4	Guselkumab	None	
5	Guselkumab	None	
6	Guselkumab	None	
7	Guselkumab	None	
8	Guselkumab	None	
9	Guselkumab	None	
10	Guselkumab	2M: conjunctival injection (discontinuation of guselkumab)	1
11	Guselkumab	None	
12	Guselkumab	None	
13	Adalimumab	2M: psoriasis-like rash (discontinuation of adalimumab)	1
14	Adalimumab	None	
15	Adalimumab	None	
16	Adalimumab	None	
17	Adalimumab	5M: psoriasis-like rash (discontinuation of adalimumab)	1
18	Adalimumab	None	
19	Adalimumab	None	
20	Adalimumab	None	
21	Adalimumab	None	
22	Adalimumab	1M: gastroenteritis	1
23	Adalimumab	None	
24	Adalimumab	4M: inadequate response (discontinuation of adalimumab)	1
25	Adalimumab	1M: psoriasis-like rash (discontinuation of adalimumab)	2

*CTCAE* National Cancer Institute Common Terminology Criteria for Adverse Events

### Impact of bDMARD treatment on peripheral immunophenotypes

Table 4 shows the results of the comprehensive peripheral blood immunophenotyping at baseline in 22 patients with PAO, 34 patients with PsA, and 30 HCs. The proportion of activated Th1 cells was significantly lower in patients with PAO than in HCs and patients with PsA. The proportion of activated Th17 cells was significantly higher in patients with PAO and PsA than in HCs. No statistically significant differences were observed in the proportion of other immune cells. We compared the proportion of activated Th1/Th17 cells at baseline in PAO patients with concomitant DMARD use (with DMARDs: *n*=16) and those without concomitant use (without DMARDs: *n*=6). No significant differences were observed between the two groups (Supplementary Table S2).

**Table 4:** Comparison in peripheral blood immune phenotypes between PAO (*n*=22), PsA (*n*=33), and HCs (*n*=30)

	HC	PsA	PAO	*p* value		
				HC vs PsA	HC vs PAO	PsA vs PAO
CD4^+^ T cells						
Naive	55.7 (43.5, 61.3)	49.2 (32.3, 60.0)	51.6 (45.0, 63.2)	0.8780	1.0000	0.9810
Central memory	34.4 (25.3, 41.7)	32.8 (24.6, 44.6)	31.5 (23.0, 37.8)	1.0000	1.0000	1.0000
Effector memory	11.1 (6.8, 15.6)	10.1 (7.4, 17.8)	10.0 (6.5, 14.9)	1.0000	1.0000	1.0000
TEMRA	2.4 (1.5, 4.0)	3.2 (2.1, 4.7)	2.5 (1.5, 4.6)	0.2980	1.0000	0.6955
Th1	17.7 (13.1, 26.0)	20.5 (17.0, 24.4)	17.8 (12.3, 19.8)	0.6549	1.0000	0.1041
Th17	9.8 (7.6, 12.8)	10.9 (9.1, 14.7)	12.1 (9.3, 16.6)	0.8311	0.1874	1.0000
Treg	4.6 (3.8, 5.6)	4.7 (3.6, 5.7)	4.3 (3.3, 5.7)	1.0000	1.0000	1.0000
Tfh	0.8 (0.7, 1.4)	0.7 (0.3, 1.4)	0.8 (0.7, 1.1)	0.6043	1.0000	0.8582
CD8^+^ T cells						
Naive	40.7 (24.9, 54.9)	44.4 (34.5, 56.2)	34.4 (29.2, 49.7)	1.0000	1.0000	0.3747
Central memory	8.2 (4.9, 28.5)	17.1 (9.7, 26.9)	19.9 (13.4, 29.3)	0.2300	0.1120	1.0000
Effector memory	12.9 (9.0, 27.5)	9.8 (5.3, 19.8)	19.8 (10.6, 24.9)	0.1757	1.0000	0.0649
TEMRA	21.7 (11.6, 29.9)	18.8 (12.0, 31.9)	17.8 (12.4, 28.6)	1.0000	1.0000	1.0000
Activated T cells						
CD4^+^	4.8 (3.1, 7.1)	4.3 (2.3, 7.1)	3.4 (2.3, 6.0)	1.0000	0.6483	1.0000
Th1	1.0 (0.6, 1.7)	1.2 (0.8, 1.6)	0.4 (0.2, 0.5)	0.7808	*<0.0001	*<0.0001
Th17	0.6 (0.4, 0.7)	1.1 (0.5, 1.5)	1.1 (0.5, 1.8)	*0.0032	*0.0164	1.0000
Treg	1.1 (1.0, 1.5)	0.9 (0.6, 1.4)	1.4 (0.7, 1.8)	0.3430	1.0000	0.2660
Tfh	0.3 (0.2, 0.5)	0.2 (0.1, 0.5)	0.3 (0.2, 0.4)	1.0000	1.0000	0.5850
CD8^+^	8.9 (5.9, 13.4)	6.8 (4.8, 13.1)	6.5 (3.8, 11.2)	0.6867	0.3812	1.0000
B cells						
Naive	61.8 (51.0, 69.4)	65.4 (58.4, 73.4)	62.0 (55.4, 68.8)	0.2550	1.0000	0.8862
IgM memory	20.8 (10.9, 25.6)	18.1 (13.8, 22.9)	16.4 (10.3, 21.1)	0.8355	0.231	1.0000
Class-switched	11.7 (8.9, 19.3)	10.6 (7.5, 15.7)	11.9 (8.2, 17.0)	0.8051	1.0000	1.0000
Double negative	5.3 (4.2, 6.6)	6.1 (4.4, 10.1)	5.1 (3.8, 7.9)	0.3217	1.0000	0.7185
Plasmocytes	2.0 (1.3, 2.9)	1.5 (0.4, 2.4)	2.5 (0.6, 4.8)	0.1329	1.0000	0.3199
Monocytes						
Classical	90.2 (82.9, 93.1)	92.3 (89.2, 94.6)	91.3 (86.1, 94.1)	0.0967	0.7704	1.0000
Non-classical	9.5 (5.0, 15.3)	7.4 (5.3, 10.7)	6.0 (4.1, 11.6)	0.6651	0.3034	1.0000
Dendritic cells						
Myeloid	82.3 (69.6, 86.6)	74.6 (61.2, 82.5)	75.5 (67.7, 85.3)	0.3641	1.0000	1.0000
Plasmacytoid	5.5 (3.8, 7.9)	6.9 (4.5, 11.5)	6.9 (4.9, 10.3)	0.2533	0.3666	1.0000
NK cells						
CD16+	94.7 (89.5, 97.0)	92.7 (88.9, 94.6)	91.8 (84.5, 95.0)	0.4355	0.1611	1.0000
CD16-	4.9 (2.9, 9.1)	6.9 (4.6, 10.0)	7.9 (3.8, 12.9)	0.2301	0.3357	1.0000

Data are shown by median (quartile). *p* values were determined by the Dunn test

*PAO* pustulotic arthro-osteitis, *PsA* psoriatic arthritis, *TEMRA* terminally differentiated effector memory cells, *Tfh* follicular helper T cells

*p<0.05: HC (*n*=30) vs PsA (*n*=33) vs PAO (*n*=22)

**Fig. 2:**
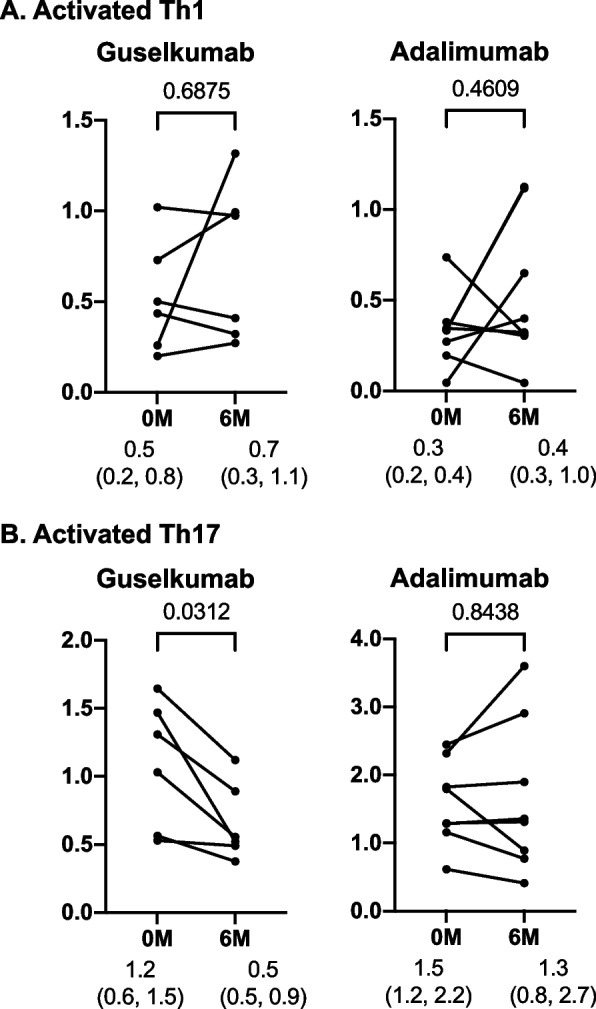
Impact of guselkumab and adalimumab treatment on activated Th1 and activated Th17 cells. The proportion of **A** activated Th1 cells to CD3^+^ and CD4^+^ T cells (%) and **B** activated Th17 cells to CD3^+^ and CD4^+^ T cells (%). *p<0.05, by Wilcoxon signed-rank test

Next, we described the changes in immunophenotypes caused by treatment (guselkumab: *n* = 6; adalimumab: *n* = 8) (Fig. 2, Supplementary Figs. S3 and S4). The proportion of activated Th1 cells did not change after treatment in either group. In contrast, the proportion of activated Th17 cells significantly decreased after treatment in the guselkumab group. Meanwhile, no significant changes were observed in the adalimumab group. No significant changes were observed in other immune cell subsets in either group. The degree of change in each immunophenotype did not correlate with the degree of change in either PPPASI or DAPSA (Supplementary Tables S3 and S4). There were no differences in the proportion of activated Th1 and Th17 cells at baseline and month 6 between DAPSA-LDA/REM responders and non-responders and PPPASI-50/75/90 responders and non-responders (Supplementary Tables S5 and S6).

### Impact of bDMARD treatment on serum cytokines

Supplementary Table S7 shows the baseline serum TNF-α and IL-17 concentrations. There were no significant differences between groups (guselkumab; *n*=12, adalimumab; *n*=11). Supplementary Fig. S5 shows changes in TNF-α and IL-17 concentration during 6 months of treatment (guselkumab; *n*=5, adalimumab; *n*=7). In both groups, significant decreases in serum TNF-α or IL-17 concentration were not observed. Focusing on the rate of decrease in cytokine concentrations, the rate of decrease in IL-17A was significantly higher in the guselkumab group (Supplementary Table S7).

## Discussion

The present study is the first to compare the safety and efficacy of guselkumab and adalimumab for the treatment of highly active PPP and simultaneously elucidate the immunological background of PAO by performing comprehensive immunophenotyping using patient samples.

In the present study, no adverse drug reactions of CTCAE grade 3 or higher occurred in either group, indicating a high safety profile for both drugs. The continuation rate in the adalimumab group was 69.2% (9/13 patients), which was lower than the continuation rate of 91.7% (11/12 patients) in the guselkumab group. Adalimumab often causes psoriasiform rash [15], which was the most common cause of discontinuation of treatment with adalimumab in the present study. In the guselkumab group, the absence of psoriasiform rash may have contributed to the high continuation rate (Table 3).

Regarding the efficacy for joint symptoms, significant improvement was observed from 1 month after treatment initiation in both groups (Tables 2 and 3). The improvement in DAPSA was comparable between the two groups (Fig. 1A, B).

This suggests that the effects on joint symptoms may be comparable between the guselkumab and adalimumab groups. Past reports on PAO have described changes in tender and swollen joint counts mainly for the evaluation of arthritis. In contrast, we used the DAPSA, which is a useful index for evaluating PsA, as a composite measure of disease activity [16]. In the present study, all patients had peripheral arthritis. Furthermore, DAPSA (SJ66, TJ68) evaluates not only sternoclavicular joint lesion characteristic of PAO but also PGA, Pain VAS, and C-reactive protein levels, and it is also easy to apply in clinical practice. However, PAO may be complicated by spinal and sacroiliac joint lesions, such as in PsA. In fact, the present study included patients with spinal or sacroiliac arthritic lesions. Therefore, DAPSA may have been an inadequate index for evaluating PAO, which exhibits diverse clinical features as described above. The present study evaluated HAQ and EQ-5D scores for PROs. These indices are based on the evaluation of the quality of life related to physical function. Since the present study showed comparable improvements in these indices, both drugs are comparable in terms of efficacy for arthritis.

As for skin symptoms, significant improvement was observed 3 months after treatment initiation in the guselkumab group, and the PPPASI reduction rate was higher in the guselkumab group than in the adalimumab group. These trends were similar to those observed in clinical studies on psoriasis [17, 18]. Although there was no statistically significant difference, the DMARD concomitant rate tended to be higher in the adalimumab group (Table 1). This difference in concomitant DMARD use might indicate the un-favorable baseline background (difficult to treat population) or disease activity had already been well controlled; therefore, this trend might contribute to the difficulty in detecting the additional effect of adalimumab treatment.

Since only guselkumab was approved for refractory PAO in Japan in November 2018, guselkumab had been administered in all cases of treatment-resistant PAO at our facility. Prior to that, adalimumab was administered for treatment-resistant PAO. For adalimumab, cases after November 2018 should be included in the study, but as described, adalimumab was not used after November 2018, to follow the regulation of the health insurance system in Japan. Therefore, we compared the guselkumab for 3 years since November 2018 and adalimumab for 3 years until November 2018. Cases (adalimumab) before 2015 were not included in this study because of concerns about differences in treatment levels other than bDMARDs. However, there is a possibility that such differences in recruitment periods could have affected the results.

The IL-23/Th17 cell axis is the primary driver of skin inflammation in psoriasis. It was reported that gene expression in psoriatic skin lesions was enhanced through IL-17 and its pathways and that treatment with guselkumab decreased the expression of IL-17-related factors and resulted in the resolution of skin lesions [19]. The present study compared the peripheral immune cell phenotypes of patients with PAO with those of HCs and patients with PsA. The results showed that the proportion of activated Th1 cells was significantly lower in patients with PAO than in HCs and patients with PsA, whereas the proportion of activated Th17 cells was significantly higher in patients with PAO (Table 4). We considered the possibility that previous (concomitant) DMARDs may have reduced activated Th1 cells or Th17 cells; however, as shown in Supplementary Table S2, no significant difference was observed between cases with concomitant DMARD use (*N*=16) and those without concomitant use (*N*=6). In other words, it was suggested that the history of DMARD use may not affect activated Th1/Th17 cells. On the other hand, the cytokine assay system used in this study detects both free cytokines and drug combined form; we could not measure the free TNF-α levels in the adalimumab. This might be one of the significant reasons that consistent changes in serum TNF-α levels were not observed in the adalimumab group. In addition, although serum samples were not thawed until cytokine measurement, cytokine levels might be influenced by the freeze/thaw cycle of the serum samples.

As described in our previous report [20], the proportion of activated Th17 cells was significantly higher in patients with PsA than in HCs; however, no significant difference was observed between the groups in the proportion of activated Th1 cells. Although this suggests that Th17 cells may play an important role in the pathogenesis of PAO as with PsA, the degree of dependence of the pathogenesis of PAO and PsA on Th1 and Th17 cells varies, and those Th17 cells might be more strongly involved in the pathogenesis of PAO. In addition, the comparison of immunophenotypes before and after treatment showed that direct inhibition of IL-23p19 by guselkumab significantly reduced the proportion of activated Th17 cells (Fig. 2). In addition, focusing on the rate of decrease in cytokine concentrations, the rate of decrease in IL-17A was significantly higher in the guselkumab group (Supplementary Table S7). Meanwhile, the results of a randomized controlled study evaluating the efficacy of ustekinumab (UST), an anti-IL12/23(p40) antibody that acts on both the IL-12-Th1 and IL-23-Th17 cell axes, for PPP have shown that the effects of UST on skin lesions are limited [12]. However, our previous report on PsA showed that although the proportion of activated Th1 cells decreased after treatment with UST, the proportion of activated Th17 cells did not change [20, 21]. This indicates that the selectivity of UST to the IL-23-Th17 cell axis may be limited. As suggested in the present study, the pathogenesis of PAO might be more dependent on activated Th17 cells than that of PsA. Thus, guselkumab, which is highly specific for the IL-23-Th17 cell axis and effective in reducing activated Th17 cells and serum IL-17 concentration, might have been highly effective in improving skin lesions. In other words, the validity of the IL-23-Th17 cell axis as a treatment target for PAO was demonstrated. However, the present study showed no significant correlation between the changes in activated Th17 cells and improvement in clinical symptoms (changes in PPPASI and DAPSA) because of the limited sample size (Supplementary Tables S2 and S3). Further verification using a larger sample size is necessary.

In contrast, treatment with adalimumab improved skin symptoms in some patients. Despite the lack of a significant difference in the rate of improvement in joint symptoms between the two groups, the rate was higher in the adalimumab group in terms of numerical values. This result suggests that in some cases, tumor necrosis factors are strongly involved in the pathogenesis.

Nevertheless, this study had some significant limitations, including a small sample size and a short observation period. We assumed that there are IL-17-dependent pathological processes in patients with higher levels of activated Th17 and that IL-17-targeting therapy, thereby, should be more effective in these patients. However, in this pilot study, we found no correlation between the proportion of activated Th17 cells and baseline disease activity or the rate of decrease in activated Th17 cells and the rate of improvement in disease activity. Similarly, it has not been verified whether adalimumab show higher efficacy in cases with fewer activated Th17 cells. Other limitations include the following: the different recruitment periods had affected the results, there is a lack of the measurement of drug concentration or anti-drug antibodies, it was possible that anti-drug antibodies were present in cases with poor treatment responses, and the adalimumab group might include more treatment-resistant cases. On the other hand, because PAO is a very rare disease, we believe that our pilot study could add new insight into PAO treatment. It is necessary for rigorous verification to conduct a prospective intervention study with a placebo group, more uniform background factors, a larger sample size, a longer observation period, and the participation of multiple institutions.

## Conclusions

Although guselkumab and adalimumab have comparable efficacy for PAO, their impact on immunophenotypes and cytokine profile vary. Based on the findings of our pilot study, it is expected to clarify the differences in the action of drugs on immunological phenotyping and cytokine profile with a larger sample size.

## Supplementary Information

**Additional file 1: Figure S1.** Flow cytometry gating strategy. The proportion of A. CD4^+^ T cells subsets to CD3^+^ and CD4^+^ T cells (%), B. CD8^+^ T cells subsets to CD3^+^ and CD8^+^ T cells (%), C. a)-e) Activated CD4^+^ T cells to CD3^+^ and CD4^+^ T cells (%) f) Activated CD8^+^ T cells to CD3^+^ and CD8^+^ T cells (%), D. B cells subsets to CD3^-^ and CD19^+^ B cells (%), E. Classical and non-classical monocytes to CD3^-^, CD19^-^, CD20^-^ and CD14^+^ cells (%), F. Myeloid and Plasmacytoid DCs to CD3^-^, CD19^-^, CD20^-^ CD14^-^ and human leukocyte antigen-DR^+^ cells (%), G. CD16+ and CD16- NK cells to CD3^-^, CD19^-^, CD20^-^ CD14^-^ and CD56^+^ cells (%).

**Additional file 2: Figure S2.** Comparison of treatment response at month 1 and 3. A. Rate of DAPSA-LDA Responders (%) B. Rate of DAPSA-REM Responders C. Rate of PPPASI-50 Responders (%) D. Rate of PPPASI-75 Responders E. Rate of PPPASI-90 Responders (%) at month q1 and 3. *p<0.05, by Fisher’s exact test. Abbreviation: PPPASI; Palmoplantar Pustulosis Area. Severity index, DAPSA; disease activity in psoriatic arthritis, LDA; low disease activity, REM; remission.

**Additional file 3: Figure S3.** Impact of guselkumab treatment on peripheral blood immune phenotypes. Changes in the proportion of A. CD4^+^ T cells subsets to CD3^+^ and CD4^+^ T cells (%), B. CD8^+^ T cells subsets to CD3^+^ and CD8^+^ T cells (%), C. a)-e) Activated CD4^+^ T cells to CD3^+^ and CD4^+^ T cells (%) f) Activated CD8^+^ T cells to CD3^+^ and CD8^+^ T cells (%), D. B cells subsets to CD3^-^ and CD19^+^ B cells (%), E. Classical and non-classical monocytes to CD3^-^, CD19^-^, CD20^-^ and CD14^+^ cells (%), F. Myeloid and Plasmacytoid DCs to CD3^-^, CD19^-^, CD20^-^ CD14^-^ and human leukocyte antigen-DR^+^ cells (%), G. CD16+ and CD16- NK cells to CD3^-^, CD19^-^, CD20^-^ CD14^-^ and CD56^+^ cells(%). *p<0.05, by Wilcoxon signed rank test.

**Additional file 4: Figure S4.** Impact of adalimumab treatment on peripheral blood immune phenotypes. Changes in the proportion of A. CD4^+^ T cells subsets to CD3^+^ and CD4^+^ T cells (%), B. CD8^+^ T cells subsets to CD3^+^ and CD8^+^ T cells (%), C. a)-c) Activated CD4^+^ T cells to CD3^+^ and CD4^+^ T cells (%) d) Activated CD8^+^ T cells to CD3^+^ and CD8^+^ T cells (%), D. B cells subsets to CD3^-^ and CD19^+^ B cells (%), E. Classical and non-classical monocytes to CD3^-^, CD19^-^, CD20^-^ and CD14^+^ cells (%), F. Myeloid and Plasmacytoid DCs to CD3^-^, CD19^-^, CD20^-^ CD14^-^ and human leukocyte antigen-DR^+^ cells (%), G. CD16+ and CD16- NK cells to CD3^-^, CD19^-^, CD20^-^ CD14^-^ and CD56^+^ cells(%). *p<0.05, by Wilcoxon signed rank test.

**Additional file 5: Figure S5.** Changes in serum TNF-α and IL-17 concentration during 6 months of treatment. TNF-α (pg/ml), B. IL-17A (fg/ml). *p<0.05, by Wilcoxon signed rank test. TNF; tumor necrosis factor.

**Additional file 6: Supplementary Table S1.** Flow cytometry antibody panels used in this study.

**Additional file 7: Supplementary Table S2.** Comparison of activated Th1 and Th17 at baseline between with DMARDs group (*N* = 16) and without DMARDs group (*N* = 6). Data are shown by median(quartile) or n (%). *P* values were determined by the Wilcoxon rank sum test. p*<0.05: with DMARDs (*N* = 16) vs without DMARDs (*N* = 6).

**Additional file 8: Supplementary Table S3.** Correlation between changes in each immunophenotype and in the PPPASI. Spearman’s rank correlation coefficient.

**Additional file 9: Supplementary Table S4.** Correlation between changes in each immunophenotype and in the DAPSA. Spearman’s rank correlation coefficient.

**Additional file 10: Supplementary Table S5.** Comparison of activated Th1 and Th17 between DAPSA-LDA/REM responder and non-responder. Data are shown by median(quartile). *P* values were determined by Wilcoxon rank sum test. p*<0.05: with DAPSA-LDA responder (*N* = 18) vs non-responder (*N* = 4) at baseline, DAPSA-REM responder (*N* = 4) vs non-responder (*N* = 18) at baseline, DAPSA-LDA responder (*N* = 11) vs non-responder (*N* = 3) at 6 months, and DAPSA-REM responder (*N* = 3) vs non-responder (*N* = 11) at 6 months.

**Additional file 11: Supplementary Table S6.** Comparison of activated Th1 and Th17 between PPPASI-50/75/90 responder and non-responder in PAO. Data are shown by median(quartile). *P* values were determined by Wilcoxon rank sum test. p*<0.05: with PPPASI-50 responder (*N* = 14) vs non-responder (*N* = 7) at baseline, PPPASI-75 responder (*N* = 13) vs non-responder (*N* = 8) at baseline, PPPASI-90 responder (*N* = 12) vs non-responder (*N* = 9) at baseline, PPPASI-50/75 responder (*N* = 7) vs non-responder (*N* = 6) at 6 months, and PPPASI-90 responder (*N* = 6) vs non-responder (*N* = 7) at months 6.

**Additional file 12: Supplementary Table S7.** Comparison of baseline cytokine concentration and their decrease rates between guselkumab group (*N* = 5) and adalimumab group (*N* = 7). Data are shown by median(quartile). *P* values were determined by Wilcoxon rank sum test. p*<0.05: with Baseline serum cytokines: guselkumab group (*N* = 12) vs adalimumab group (*N* = 10), Decrease rates of cytokines: guselkumab group (*N* = 5) vs adalimumab group (*N* = 7). PsA; Psoriatic arthritis, PAO; pustulotic arthro-osteitis.

## Data Availability

The data are available from the corresponding author (YT), upon reasonable request.

## Abbreviations

GUS: Guselkumab
ADA: Adalimumab
PAO: Pustulotic arthro-osteitis
PROs: Patient-reported outcomes
Th: T helper
PPP: Palmoplantar pustulosis
TNF: Tumor necrosis factor
IL: Interleukin
SJ: Swollen joint
TJ: Tender joint
PGA: Patient Global Assessment
Pain VAS: Pain Visual Analog Scale
DAPSA: Disease activity in psoriatic arthritis
PPPASI: Palmoplantar Pustulosis Area Severity Index
HAQ: Health Assessment Questionnaire
EQ-5D: European Quality of Life Score with five dimensions
LDA: Low disease activity
REM: Remission
CTCAE: National Cancer Institute Common Terminology Criteria for Adverse Events
HCs: Healthy controls
PsA: Psoriatic arthritis
DCs: Dendritic cells
