# Correction: Efficacy and safety of guselkumab and adalimumab for pustulotic arthro-osteitis and their impact on peripheral blood immunophenotypes

**DOI:** 10.1186/s13075-023-03005-x

**Published:** 2023-02-14

**Authors:** Masanobu Ueno, Ippei Miyagawa, Yusuke Miyazaki, Kentaro Hanami, Shunsuke Fukuyo, Satoshi Kubo, Shingo Nakayamada, Yoshiya Tanaka

**Affiliations:** 1 The First Department of Internal Medicine, School of Medicine, University of Occupational and Environmental Health, Japan, 1-1 Iseigaoka, Kitakyushu, 807-8555 Japan; 2 Department of Internal Medicine 3, Rheumatology and Immunology, Friedrich-Alexander-University Erlangen-Nurnberg (FAU) and Universitatsklinikum Erlangen, Erlangen, Germany; 3 Deutsches Zentrum fur Immuntherapie (DZI), FriedrichAlexander-University Erlangen-Nurnberg (FAU) and Universitatsklinikum Erlangen, Erlangen, Germany

**Correction: Arthritis Res Ther 24, 240 (2022)**

**https://doi.org/10.1186/s13075-022-02934-3**

Following publication of the original article [1], the authors reported an error in Additional file 1: Figure S1, Panel (C), subpanel c) a description regarding the gating strategy for the activated Th17 cells.

The data currently reads:

- Activated Th17 cells: CD3^+^, CD4^+^, CXCR3^+^, CCR6^+^, CD38^+^, HLA-DR^+^

The data should read:

- Activated Th17 cells: CD3^+^, CD4^+^, CXCR3^-^, CCR6^+^, CD38^+^, HLA-DR^+^

## Supplementary Information

**Additional file 1: Figure S1.** Flow cytometry gating strategy. The proportion of A. CD4^+^ T cells subsets to CD3^+^ and CD4^+^ T cells (%), B. CD8^+^ T cells subsets to CD3^+^ and CD8^+^ T cells (%), C. a)-e) Activated CD4^+^ T cells to CD3^+^ and CD4^+^ T cells (%) f) Activated CD8^+^ T cells to CD3^+^ and CD8^+^ T cells (%), D. B cells subsets to CD3^-^ and CD19^+^ B cells (%), E. Classical and non-classical monocytes to CD3^-^, CD19^-^, CD20^-^ and CD14^+^ cells (%), F. Myeloid and Plasmacytoid DCs to CD3^-^, CD19^-^, CD20^-^ CD14^-^ and human leukocyte antigen-DR^+^ cells (%), G. CD16+ and CD16- NK cells to CD3^-^, CD19^-^, CD20^-^ CD14^-^ and CD56^+^ cells (%).
