# Supplementary material for: Efficacy and safety of guselkumab and adalimumab for pustulotic arthro-osteitis and their impact on peripheral blood immunophenotypes
Source: Arthritis Res Ther. 2022 Oct 27;24:240. doi: 10.1186/s13075-022-02934-3 (PMC9609190; doi:10.1186/s13075-022-02934-3)

Supplementary Figure S1

(A) CD4$^+$ T cells

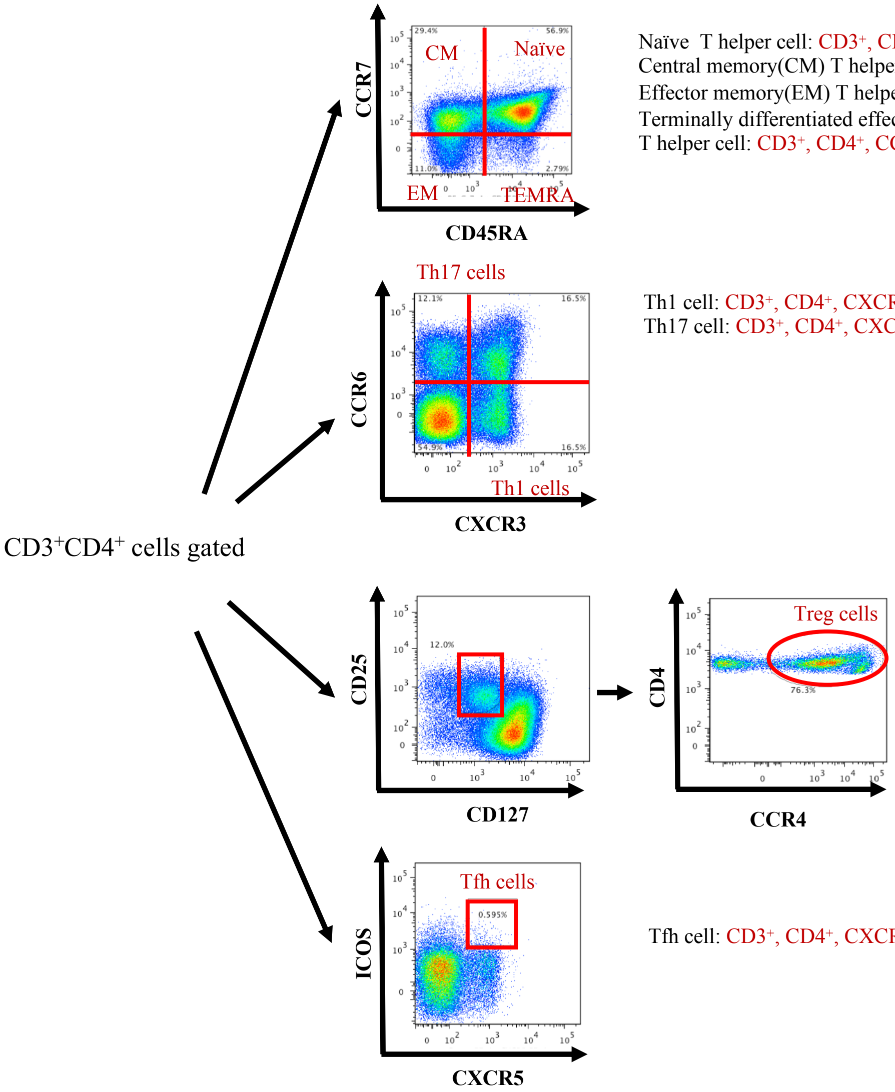

Naïve T helper cell: CD3$^+$, CD4$^+$, CCR7$^+$, CD45RA$^+$
Central memory(CM) T helper cell: CD3$^+$, CD4$^+$, CCR7$^+$, CD45RA$^-$
Effector memory(EM) T helper cell: CD3$^+$, CD4$^+$, CCR7$^-$, CD45RA$^-$
Terminally differentiated effector memory(TEMRA) T helper cell: CD3$^+$, CD4$^+$, CCR7$^-$, CD45RA$^+$

Th1 cell: CD3$^+$, CD4$^+$, CXCR3$^+$, CCR6$^-$
Th17 cell: CD3$^+$, CD4$^+$, CXCR3$^-$, CCR6$^+$

Treg cell: CD3$^+$, CD4$^+$, CD25$^+$, CD127^{low}, CCR4$^+$

Tfh cell: CD3$^+$, CD4$^+$, CXCR5$^+$, ICOS$^+$

(B) CD8$^+$ T cells

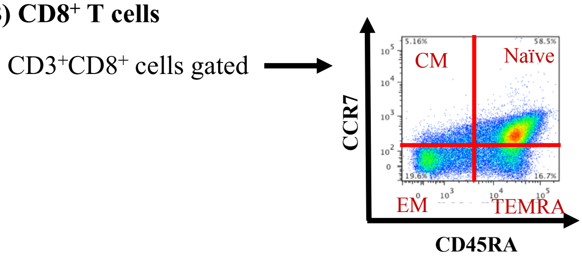

Naive cytotoxic T cell: CD3$^+$, CD8$^+$, CCR7$^+$, CD45RA$^+$
Central memory cytotoxic T cell: CD3$^+$, CD8$^+$, CCR7$^+$, CD45RA$^-$
Effector memory cytotoxic T cell : CD3$^+$, CD4$^+$, CCR7$^-$, CD45RA$^-$
Terminally differentiated effector memory cytotoxic T cell : CD3$^+$, CD4$^+$, CCR7$^-$, CD45RA$^+$

Supplementary Figure S1

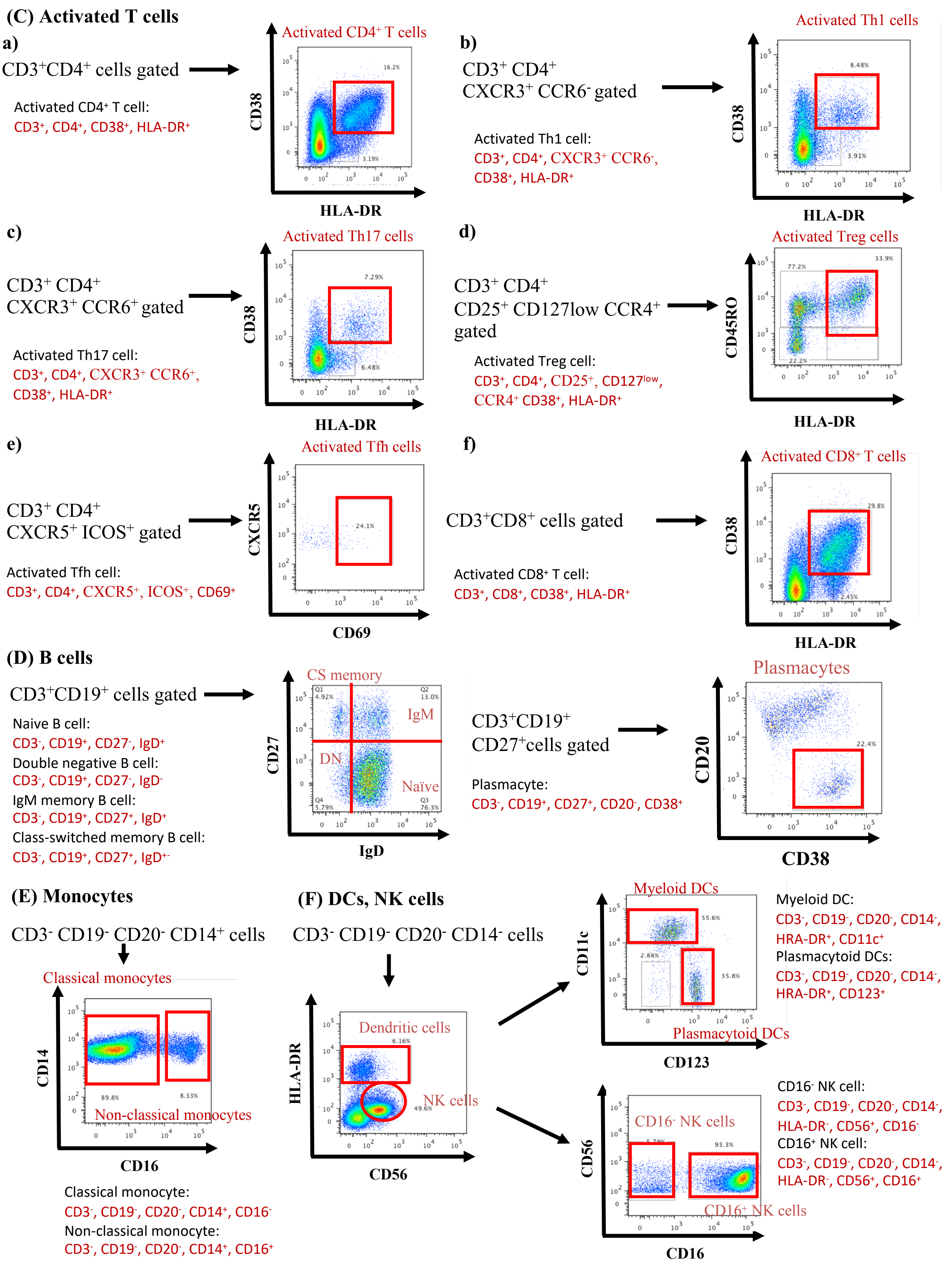

Supplement: Supplementary file 1 — Additional file 1: Figure S1. Flow cytometry gating strategy. The proportion of A. CD4+ T cells subsets to CD3+ and CD4+ T cells (%), B. CD8+ T cells subsets to CD3+ and CD8+ T cells (%), C. a)-e) Activated CD4+ T cells to CD3+ and CD4+ T cells (%) f) Activated CD8+ T cells to CD3+ and CD8+ T cells (%), D. B cells subsets to CD3- and CD19+ B cells (%), E. Classical and non-classical monocytes to CD3-, CD19-, CD20- and CD14+ cells (%), F. Myeloid and Plasmacytoid DCs to CD3-, CD19-, CD20- CD14- and human leukocyte antigen-DR+ cells (%), G. CD16+ and CD16- NK cells to CD3-, CD19-, CD20- CD14- and CD56+ cells (%). [file 13075_2022_2934_MOESM1_ESM.pdf]
