# Supplementary material for: Efficacy and safety of guselkumab and adalimumab for pustulotic arthro-osteitis and their impact on peripheral blood immunophenotypes
Source: Arthritis Res Ther. 2022 Oct 27;24:240. doi: 10.1186/s13075-022-02934-3 (PMC9609190; doi:10.1186/s13075-022-02934-3)

Supplementary Figure S2

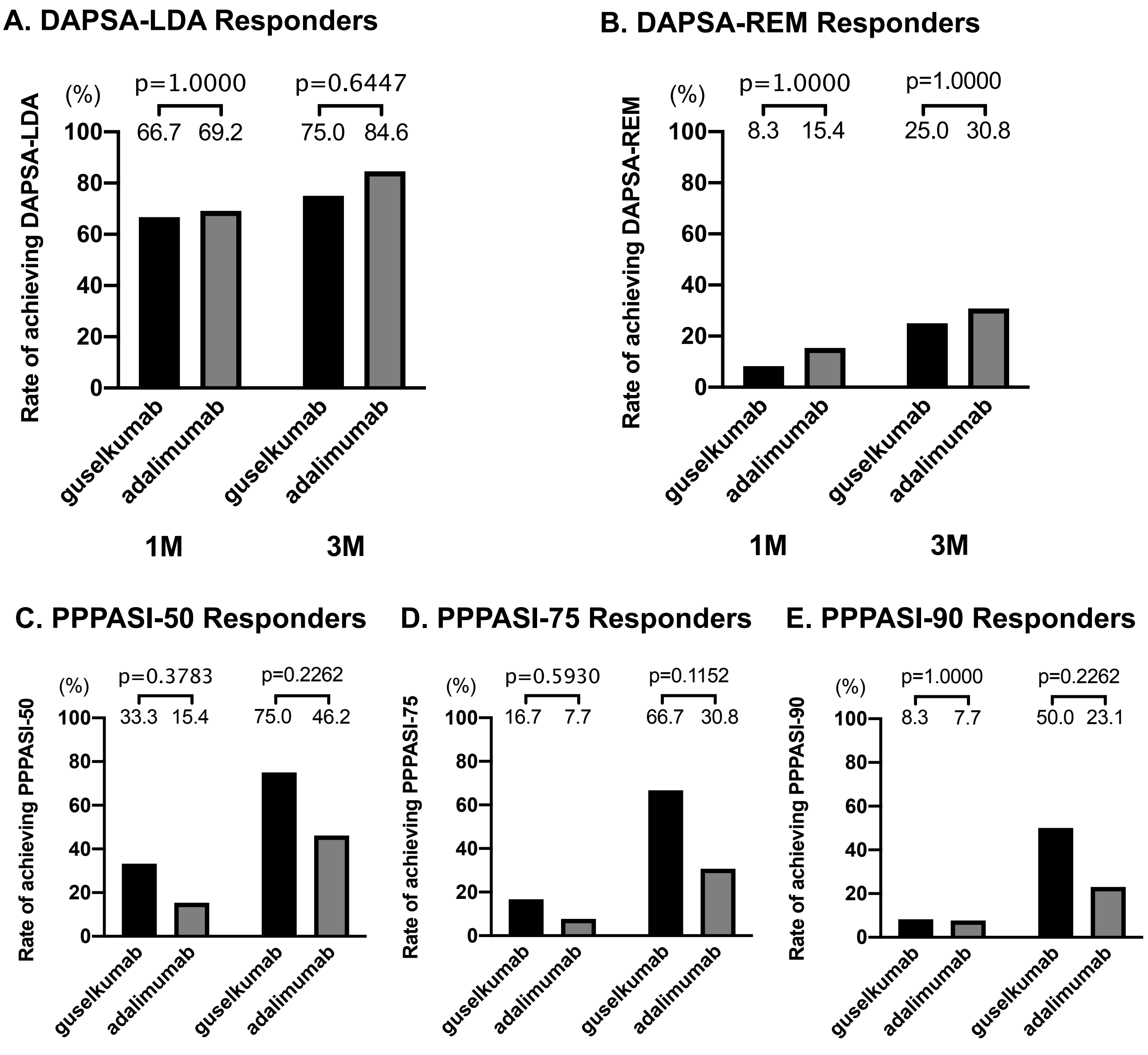

Supplement: Supplementary file 2 — Additional file 2: Figure S2. Comparison of treatment response at month 1 and 3. A. Rate of DAPSA-LDA Responders (%) B. Rate of DAPSA-REM Responders C. Rate of PPPASI-50 Responders (%) D. Rate of PPPASI-75 Responders E. Rate of PPPASI-90 Responders (%) at month q1 and 3. *p<0.05, by Fisher’s exact test. Abbreviation: PPPASI; Palmoplantar Pustulosis Area. Severity index, DAPSA; disease activity in psoriatic arthritis, LDA; low disease activity, REM; remission. [file 13075_2022_2934_MOESM2_ESM.pdf]
