# Supplementary material for: Efficacy and safety of guselkumab and adalimumab for pustulotic arthro-osteitis and their impact on peripheral blood immunophenotypes
Source: Arthritis Res Ther. 2022 Oct 27;24:240. doi: 10.1186/s13075-022-02934-3 (PMC9609190; doi:10.1186/s13075-022-02934-3)

**Supplementary Figure S4**

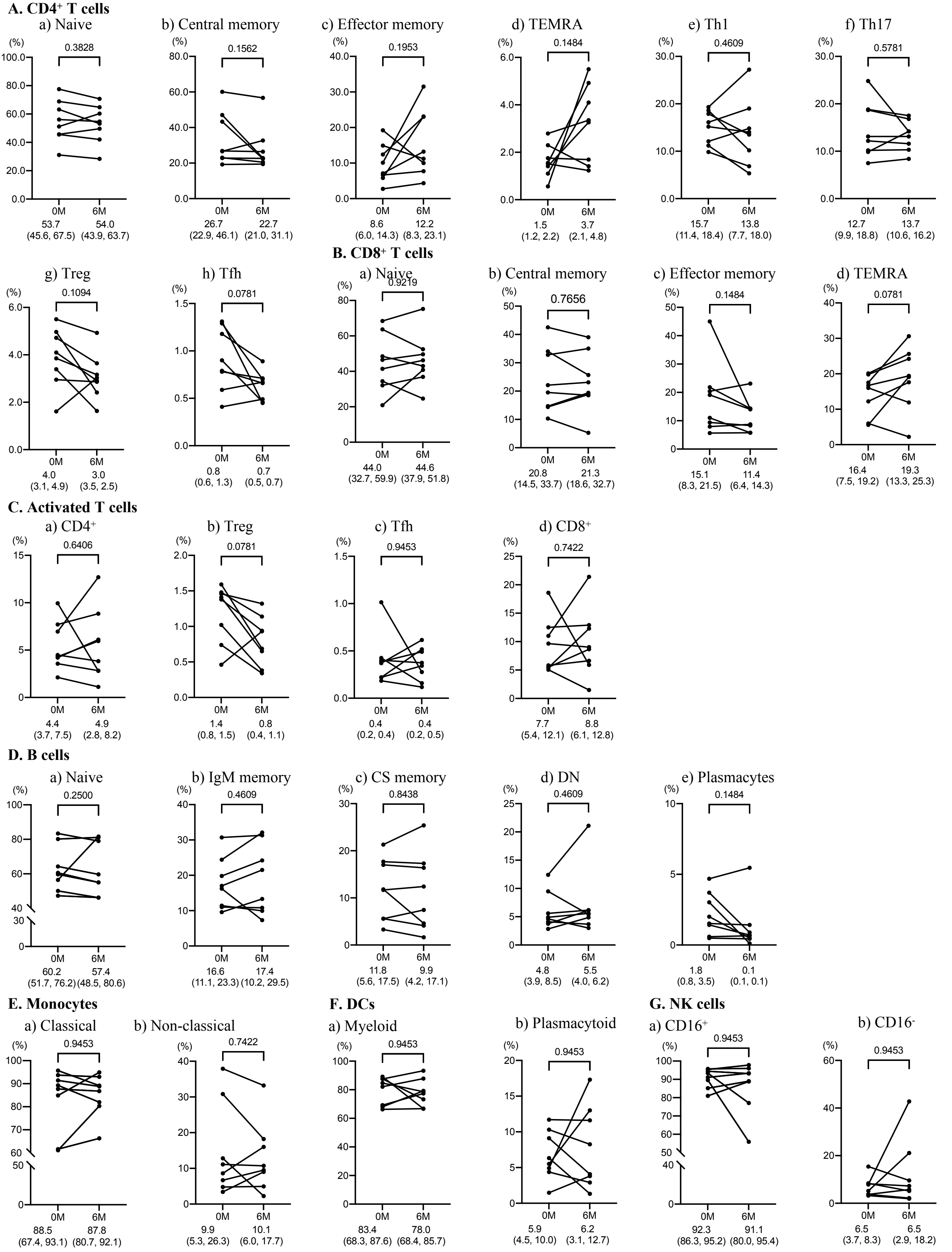

Supplement: Supplementary file 4 — Additional file 4: Figure S4. Impact of adalimumab treatment on peripheral blood immune phenotypes. Changes in the proportion of A. CD4+ T cells subsets to CD3+ and CD4+ T cells (%), B. CD8+ T cells subsets to CD3+ and CD8+ T cells (%), C. a)-c) Activated CD4+ T cells to CD3+ and CD4+ T cells (%) d) Activated CD8+ T cells to CD3+ and CD8+ T cells (%), D. B cells subsets to CD3- and CD19+ B cells (%), E. Classical and non-classical monocytes to CD3-, CD19-, CD20- and CD14+ cells (%), F. Myeloid and Plasmacytoid DCs to CD3-, CD19-, CD20- CD14- and human leukocyte antigen-DR+ cells (%), G. CD16+ and CD16- NK cells to CD3-, CD19-, CD20- CD14- and CD56+ cells(%). *p<0.05, by Wilcoxon signed rank test. [file 13075_2022_2934_MOESM4_ESM.pdf]
