# Supplementary figures and images for: Efficacy and safety of guselkumab and adalimumab for pustulotic arthro-osteitis and their impact on peripheral blood immunophenotypes
Source: Arthritis Res Ther. 2022 Oct 27;24:240. doi: 10.1186/s13075-022-02934-3 (PMC9609190; doi:10.1186/s13075-022-02934-3)

Supplementary Figure S5

a). TNF- $\alpha$

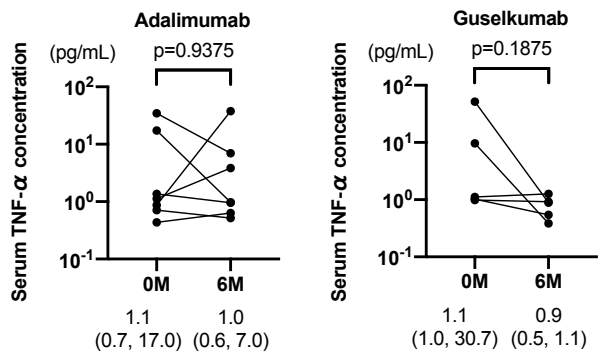

b). IL-17A

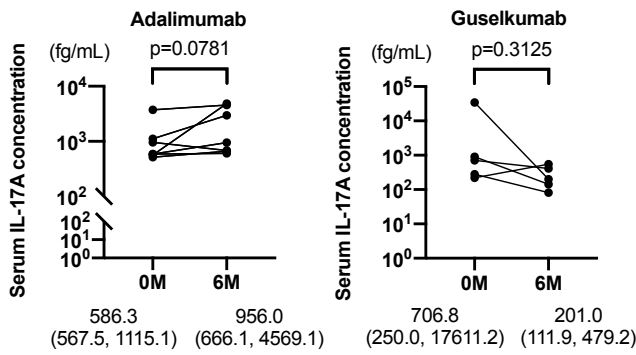

Supplement: Supplementary file 5 — Additional file 5: Figure S5. Changes in serum TNF-α and IL-17 concentration during 6 months of treatment. TNF-α (pg/ml), B. IL-17A (fg/ml). *p<0.05, by Wilcoxon signed rank test. TNF; tumor necrosis factor. [file 13075_2022_2934_MOESM5_ESM.pdf]
