# Supplementary material for: Efficacy and safety of guselkumab and adalimumab for pustulotic arthro-osteitis and their impact on peripheral blood immunophenotypes
Source: Arthritis Res Ther. 2022 Oct 27;24:240. doi: 10.1186/s13075-022-02934-3 (PMC9609190; doi:10.1186/s13075-022-02934-3)
Supplement: Supplementary file 6 — Additional file 6: Supplementary Table S1. Flow cytometry antibody panels used in this study. [file 13075_2022_2934_MOESM6_ESM.docx]

|  | **T cells** | **Treg** | **Th1, Th17** | **Tfh** | **B cells** | **DCs, NK cells Monocytes** |
| --- | --- | --- | --- | --- | --- | --- |
| **FITC** | Live or dead | Live or dead | Live or dead | CD28 | Live or dead | Live or dead |
| **PE** | CCR7 | CD25 | CXCR3 | CXCR5 | CD24 | CD56 |
| **PerCP-Cy5.5** | CD4 | CD4 | CD4 | CXCR3 | CD19 | CD123 |
| **PE-Cy7** | CD45RA | CCR4 | CCR6 | CCR6 | CD27 | CD11c |
| **APC** | CD38 | CD127 | CD38 | ICOS | CD38 | CD16 |
| **APC-H7** | CD8 | CD45RO | CD8 | CD69 | CD20 | CD3, CD19, CD20 |
| **V450** | CD3 | CD3 | CD3 | CD3 | CD3 | CD14 |
| **V500** | HLA-DR | HLA-DR | HLA-DR | CD4 | IgD | HLA-DR |

**Supplementary table S1. Flow cytometry antibody panels used in this study.**
