# Supplementary material for: Efficacy and safety of guselkumab and adalimumab for pustulotic arthro-osteitis and their impact on peripheral blood immunophenotypes
Source: Arthritis Res Ther. 2022 Oct 27;24:240. doi: 10.1186/s13075-022-02934-3 (PMC9609190; doi:10.1186/s13075-022-02934-3)
Supplement: Supplementary file 7 — Additional file 7: Supplementary Table S2. Comparison of activated Th1 and Th17 at baseline between with DMARDs group (N = 16) and without DMARDs group (N = 6). Data are shown by median(quartile) or n (%). P values were determined by the Wilcoxon rank sum test. p*<0.05: with DMARDs (N = 16) vs without DMARDs (N = 6). [file 13075_2022_2934_MOESM7_ESM.docx]

|  |  | with DMARDs (N=16) | without DMARDs (N=6) | p value |
| --- | --- | --- | --- | --- |
| Activated T cells | Th1 | 0.4 (0.3, 0.5) | 0.4 (0.2, 0.6) | 0.9262 |
|  | Th17 | 0.5 (0.3, 1.2) | 1.2 (0.5, 1.7) | 0.0956 |

**Supplementary table S2. Comparison of activated Th1 and Th17 at baseline between with DMARDs group (N=16) and without DMARDs group (N=6).**

Data are shown by median(quartile) or n (%). *P* values were determined by the Wilcoxon rank sum test. p*<0.05: with DMARDs(N=16) vs without DMARDs(N=6).
