# Supplementary material for: Efficacy and safety of guselkumab and adalimumab for pustulotic arthro-osteitis and their impact on peripheral blood immunophenotypes
Source: Arthritis Res Ther. 2022 Oct 27;24:240. doi: 10.1186/s13075-022-02934-3 (PMC9609190; doi:10.1186/s13075-022-02934-3)
Supplement: Supplementary file 8 — Additional file 8: Supplementary Table S3. Correlation between changes in each immunophenotype and in the PPPASI. Spearman’s rank correlation coefficient. [file 13075_2022_2934_MOESM8_ESM.docx]

|  |  | **correlation coefficient** | **p value** |
| --- | --- | --- | --- |
| **CD4^+^ T cells** | Naive | -0.2703 | 0.3499 |
|  | Central memory | 0.2088 | 0.4738 |
|  | Effector memory | 0.0505 | 0.8637 |
|  | TEMRA | 0.389 | 0.1692 |
|  | Th1 | 0.0374 | 0.8991 |
|  | Th17 | -0.3542 | 0.214 |
|  | Treg | 0.1297 | 0.6586 |
|  | Tfh | -0.0593 | 0.8403 |
| **CD8^+^ T cells** | Naive | 0.2352 | 0.4183 |
|  | Central memory | -0.0066 | 0.9822 |
|  | Effector memory | 0.033 | 0.9109 |
|  | TEMRA | -0.0374 | 0.8991 |
| **Activated T cells** | CD4^+^ | 0.0593 | 0.8403 |
|  | Th1 | 0.1209 | 0.6806 |
|  | Th17 | 0.0242 | 0.9346 |
|  | Treg | -0.0418 | 0.8873 |
|  | Tfh | 0.4202 | 0.1346 |
|  | CD8^+^ | 0.3451 | 0.2269 |
| **B cells** | Naive | 0.1429 | 0.6261 |
|  | IgM memory | -0.1165 | 0.6917 |
|  | Class-switched | -0.1341 | 0.6477 |
|  | Double negative | 0.156 | 0.5942 |
|  | Plasmocytes | -0.0681 | 0.817 |
| **Monocytes** | Classical | -0.2571 | 0.3748 |
|  | Non-classical | 0.1912 | 0.5126 |
| **Dendritic cells** | Myeloid | 0.275 | 0.3413 |
|  | Plasmacytoid | -0.2132 | 0.4643 |
| **NK cells** | CD16+ | 0.1033 | 0.7253 |
|  | CD16- | -0.1604 | 0.5838 |

**Supplementary table S3. Correlation between changes in each immunophenotype and in the PPPASI.** Spearman’s rank correlation coefficient
