# Supplementary material for: Efficacy and safety of guselkumab and adalimumab for pustulotic arthro-osteitis and their impact on peripheral blood immunophenotypes
Source: Arthritis Res Ther. 2022 Oct 27;24:240. doi: 10.1186/s13075-022-02934-3 (PMC9609190; doi:10.1186/s13075-022-02934-3)
Supplement: Supplementary file 9 — Additional file 9: Supplementary Table S4. Correlation between changes in each immunophenotype and in the DAPSA. Spearman’s rank correlation coefficient. [file 13075_2022_2934_MOESM9_ESM.docx]

|  |  | correlation coefficient | p value |
| --- | --- | --- | --- |
| CD4^+^ T cells | Naive | 0.252 | 0.3816 |
|  | Central memory | -0.2659 | 0.3581 |
|  | Effector memory | -0.1824 | 0.5325 |
|  | TEMRA | -0.3626 | 0.2026 |
|  | Th1 | 0.0901 | 0.7593 |
|  | Th17 | 0.4708 | 0.0893 |
|  | Treg | 0.4022 | 0.1540 |
|  | Tfh | -0.4813 | 0.0814 |
| CD8^+^ T cells | Naive | -0.2044 | 0.4833 |
|  | Central memory | 0.3363 | 0.2398 |
|  | Effector memory | -0.3011 | 0.2955 |
|  | TEMRA | -0.3978 | 0.1590 |
| Activated T cells | CD4^+^ | -0.1516 | 0.6048 |
|  | Th1 | 0.2220 | 0.4456 |
|  | Th17 | -0.3659 | 0.2192 |
|  | Treg | 0.0505 | 0.8637 |
|  | Tfh | 0.5083 | 0.0635 |
|  | CD8^+^ | 0.3407 | 0.2333 |
| B cells | Naive | -0.1648 | 0.5733 |
|  | IgM memory | 0.2440 | 0.4006 |
|  | Class-switched | -0.2659 | 0.3581 |
|  | Double negative | -0.3055 | 0.2882 |
|  | Plasmocytes | 0.1253 | 0.6696 |
| Monocytes | Classical | 0.0549 | 0.8520 |
|  | Non-classical | -0.0681 | 0.8170 |
| Dendritic cells | Myeloid | 0.4576 | 0.0999 |
|  | Plasmacytoid | -0.2527 | 0.3833 |
| NK cells | CD16+ | 0.0989 | 0.7366 |
|  | CD16- | -0.1033 | 0.7253 |

**Supplementary table S4. Correlation between changes in each immunophenotype and in the DAPSA.** Spearman’s rank correlation coefficient.
