# Supplementary material for: Efficacy and safety of guselkumab and adalimumab for pustulotic arthro-osteitis and their impact on peripheral blood immunophenotypes
Source: Arthritis Res Ther. 2022 Oct 27;24:240. doi: 10.1186/s13075-022-02934-3 (PMC9609190; doi:10.1186/s13075-022-02934-3)
Supplement: Supplementary file 10 — Additional file 10: Supplementary Table S5. Comparison of activated Th1 and Th17 between DAPSA-LDA/REM responder and non-responder. Data are shown by median(quartile). P values were determined by Wilcoxon rank sum test. p*<0.05: with DAPSA-LDA responder (N = 18) vs non-responder (N = 4) at baseline, DAPSA-REM responder (N = 4) vs non-responder (N = 18) at baseline, DAPSA-LDA responder (N = 11) vs non-responder (N = 3) at 6 months, and DAPSA-REM responder (N = 3) vs non-responder (N = 11) at 6 months. [file 13075_2022_2934_MOESM10_ESM.docx]

|  | **DAPSA-LDA** | |  | **DAPSA-REM** | |  |
| --- | --- | --- | --- | --- | --- | --- |
|  | responder (N=18) | non-resonder (N=4) | p value | responder (N=4) | non-resonder (N=18) | p value |
| **Activated Th1 at baseline (%)** | 0.4 (0.3, 0.5) | 0.5 (0.1, 0.9) | 0.9321 | 0.4 (0.2, 0.7) | 0.3 (0.2, 0.5) | 0.6396 |
| **Activated Th17 at baseline (%)** | 1.1 (0.5, 1.8) | 1.1 (0.5, 1.5) | 0.6703 | 0.8 (0.2, 1.7) | 1.1 (0.5, 1.8) | 0.3713 |
|  | responder (N=11) | non-resonder (N=3) |  | responder (N=3) | non-resonder (N=11) |  |
| **Activated Th1 at Month 6 (%)** | 0.4 (0.3, 1.0) | 0.6 (0.3, 1.0) | 0.5325 | 0.6 (0.3, 1.0) | 0.4 (0.3, 0.9) | 0.9271 |
| **Activated Th17 at Month 6 (%)** | 1.1 (0.5, 1.9) | 0.9 (0.8, 1.1) | 0.6961 | 1.2 (1.1, 1.3) | 0.9 (0.5, 1.8) | 0.5214 |

**Supplementary table S5. Comparison of activated Th1 and Th17 between DAPSA-LDA/REM responder and non-responder.**

Data are shown by median(quartile). *P* values were determined by Wilcoxon rank sum test. p*<0.05: with DAPSA-LDA responder (N=18) vs non-responder (N=4) at baseline, DAPSA-REM responder (N=4) vs non-responder (N=18) at baseline, DAPSA-LDA responder (N=11) vs non-responder (N=3) at 6 months, and DAPSA-REM responder (N=3) vs non-responder (N=11) at 6 months.
