# Supplementary material for: Efficacy and safety of guselkumab and adalimumab for pustulotic arthro-osteitis and their impact on peripheral blood immunophenotypes
Source: Arthritis Res Ther. 2022 Oct 27;24:240. doi: 10.1186/s13075-022-02934-3 (PMC9609190; doi:10.1186/s13075-022-02934-3)
Supplement: Supplementary file 11 — Additional file 11: Supplementary Table S6. Comparison of activated Th1 and Th17 between PPPASI-50/75/90 responder and non-responder in PAO. Data are shown by median(quartile). P values were determined by Wilcoxon rank sum test. p*<0.05: with PPPASI-50 responder (N = 14) vs non-responder (N = 7) at baseline, PPPASI-75 responder (N = 13) vs non-responder (N = 8) at baseline, PPPASI-90 responder (N = 12) vs non-responder (N = 9) at baseline, PPPASI-50/75 responder (N = 7) vs non-responder (N = 6) at 6 months, and PPPASI-90 responder (N = 6) vs non-responder (N = 7) at months 6. [file 13075_2022_2934_MOESM11_ESM.docx]

|  | **PPPASI-50** | |  | **PPPASI-75** | |  | | **PPPASI-90** | | |  | |  |
| --- | --- | --- | --- | --- | --- | --- | --- | --- | --- | --- | --- | --- | --- |
|  | responder  (N=14) | non-resonder  (N=7) | p value | responder  (N=13) | non-resonder  (N=8) | | p value | | responder (N=12) | non-resonder (N=9) | | p value | |
| **Activated Th1 at baseline (%)** | 0.4 (0.2, 0.5) | 0.3 (0.2, 0.4) | 0.6015 | 0.3 (0.2, 0.5) | 0.4 (0.2, 0.4) | | 0.6639 | | 0.3 (0.2, 0.5) | 0.4 (0.2, 0.5) | | 1.0000 | |
| **Activated Th17 at baseline (%)** | 0.7 (0.4, 1.7) | 1.4 (0.6, 1.8) | 0.2631 | 0.9 (0.4, 1.7) | 1.2 (0.5, 1.8) | | 0.4689 | | 0.7 (0.3, 1.8) | 1.3 (0.6, 1.8) | | 0.3556 | |
|  | responder  (N=7) | non-resonder  (N=6) | p value | responder  (N=7) | non-resonder  (N=6) | | p value | | responder (N=6) | non-resonder (N=7) | | p value | |
| **Activated Th1 at Month 6 (%)** | 0.4 (0.3, 1.0) | 0.5 (0.2, 1.0) | 0.9429 | 0.4 (0.3, 1.0) | 0.5 (0.2, 1.0) | | 0.9429 | | 0.4 (0.3, 1.0) | 0.4 (0.3, 1.0) | | 1.0000 | |
| **Activated Th17 at Month 6 (%)** | 0.9 (0.5, 1.9) | 1.2 (0.7, 1.7) | 0.6161 | 0.9 (0.5, 1.9) | 1.2 (0.7, 1.7) | | 0.6161 | | 1.0 (0.5, 2.3) | 1.1 (0.5, 1.4) | | 1.0000 | |

**Supplementary table S6. Comparison of activated Th1 and Th17 between PPPASI-50/75/90 responder and non-responder in PAO.**

Data are shown by median(quartile). *P* values were determined by Wilcoxon rank sum test. p*<0.05: with PPPASI-50 responder (N=14) vs non-responder (N=7) at baseline, PPPASI-75 responder (N=13) vs non-responder (N=8) at baseline, PPPASI-90 responder (N=12) vs non-responder (N=9) at baseline, PPPASI-50/75 responder (N=7) vs non-responder (N=6) at 6 months, and PPPASI-90 responder (N=6) vs non-responder (N=7) at months 6.
