# Supplementary material for: Efficacy and safety of guselkumab and adalimumab for pustulotic arthro-osteitis and their impact on peripheral blood immunophenotypes
Source: Arthritis Res Ther. 2022 Oct 27;24:240. doi: 10.1186/s13075-022-02934-3 (PMC9609190; doi:10.1186/s13075-022-02934-3)
Supplement: Supplementary file 12 — Additional file 12: Supplementary Table S7. Comparison of baseline cytokine concentration and their decrease rates between guselkumab group (N = 5) and adalimumab group (N = 7). Data are shown by median(quartile). P values were determined by Wilcoxon rank sum test. p*<0.05: with Baseline serum cytokines: guselkumab group (N = 12) vs adalimumab group (N = 10), Decrease rates of cytokines: guselkumab group (N = 5) vs adalimumab group (N = 7). PsA; Psoriatic arthritis, PAO; pustulotic arthro-osteitis. [file 13075_2022_2934_MOESM12_ESM.docx]

| **Baseline serum cytokines** | **guselkumab group (N=12)** | **adalimumab group (N=10)** | **p value** |
| --- | --- | --- | --- |
| TNF-α (pg/mL) | 5.0 (1.0, 13.9) | 0.9 (0.6, 5.4) | 0.1294 |
| IL-17A (fg/mL) | 839.2 (376.4, 1322.8) | 607.2 (554.4, 1295.9) | 0.5528 |
| **Changes in cytokine concentrations (%)** | **guselkumab group (N=5)** | **adalimumab group (N=7)** | **p value** |
| TNF-α | -46.5 (-97.1, +3.3) | -27.1 (-79.7, +243.3) | 0.2232 |
| IL-17A | -70.9 (-91.8, +53.2) | +29.3 (+3.7, +166.8) | *0.0424 |

**Supplementary table S7. Comparison of baseline cytokine concentration and their decrease rates between guselkumab group(N=5) and adalimumab group(N=7).**

Data are shown by median(quartile). *P* values were determined by Wilcoxon rank sum test. p*<0.05: with Baseline serum cytokines: guselkumab group(N=12) vs adalimumab group(N=10), Decrease rates of cytokines: guselkumab group(N=5) vs adalimumab group(N=7).

PsA; Psoriatic arthritis, PAO; pustulotic arthro-osteitis.
