# Supplementary material for: Correction: Efficacy and safety of guselkumab and adalimumab for pustulotic arthro-osteitis and their impact on peripheral blood immunophenotypes
Source: Arthritis Res Ther. 2023 Feb 14;25:24. doi: 10.1186/s13075-023-03005-x (PMC9926818; doi:10.1186/s13075-023-03005-x)
Supplement: Supplementary file 1 — Additional file 1: Figure S1. Flow cytometry gating strategy. The proportion of A. CD4+ T cells subsets to CD3+ and CD4+ T cells (%), B. CD8+ T cells subsets to CD3+ and CD8+ T cells (%), C. a)-e) Activated CD4+ T cells to CD3+ and CD4+ T cells (%) f) Activated CD8+ T cells to CD3+ and CD8+ T cells (%), D. B cells subsets to CD3- and CD19+ B cells (%), E. Classical and non-classical monocytes to CD3-, CD19-, CD20- and CD14+ cells (%), F. Myeloid and Plasmacytoid DCs to CD3-, CD19-, CD20- CD14- and human leukocyte antigen-DR+ cells (%), G. CD16+ and CD16- NK cells to CD3-, CD19-, CD20- CD14- and CD56+ cells (%). [file 13075_2023_3005_MOESM1_ESM.pptx]

## Slide 1
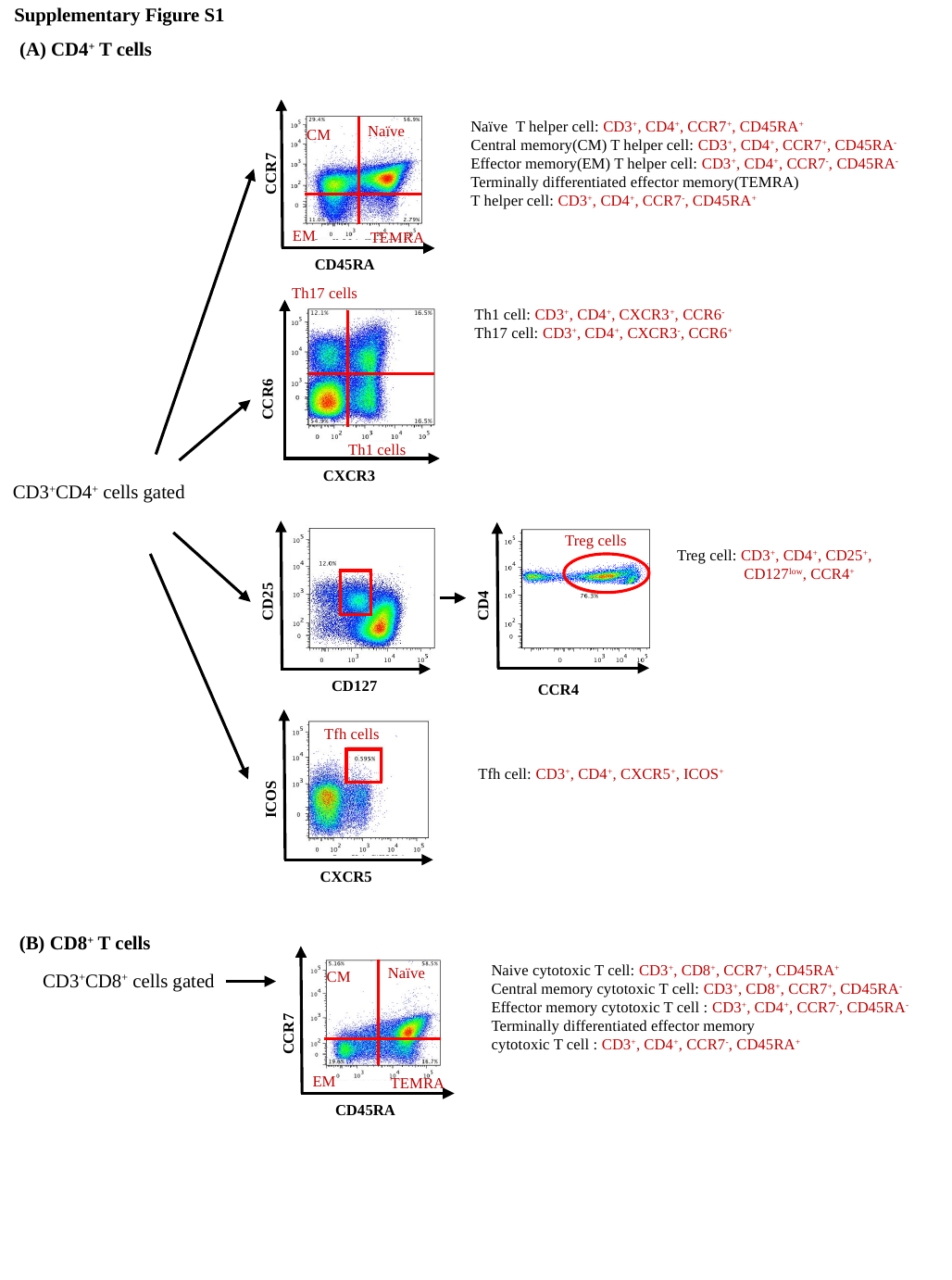

Supplementary Figure S1
(A) CD4+ T cells
Naïve
CM
CCR7
EM
TEMRA
CD45RA
Naïve T helper cell: CD3+, CD4+, CCR7+, CD45RA+
Central memory(CM) T helper cell: CD3+, CD4+, CCR7+, CD45RA-
Effector memory(EM) T helper cell: CD3+, CD4+, CCR7-, CD45RA-
Terminally differentiated effector memory(TEMRA)
T helper cell: CD3+, CD4+, CCR7-, CD45RA+
Th17 cells
CCR6
Th1 cells
CXCR3
Th1 cell: CD3+, CD4+, CXCR3+, CCR6-
Th17 cell: CD3+, CD4+, CXCR3-, CCR6+
CD3+CD4+ cells gated
CD25
CD127
Treg cells
CD4
CCR4
Treg cell: CD3+, CD4+, CD25+,
 CD127low, CCR4+
Tfh cells
ICOS
CXCR5
Tfh cell: CD3+, CD4+, CXCR5+, ICOS+
(B) CD8+ T cells
Naïve
CM
CCR7
EM
TEMRA
CD45RA
Naive cytotoxic T cell: CD3+, CD8+, CCR7+, CD45RA+
Central memory cytotoxic T cell: CD3+, CD8+, CCR7+, CD45RA-
Effector memory cytotoxic T cell : CD3+, CD4+, CCR7-, CD45RA-
Terminally differentiated effector memory
cytotoxic T cell : CD3+, CD4+, CCR7-, CD45RA+
CD3+CD8+ cells gated

## Slide 2
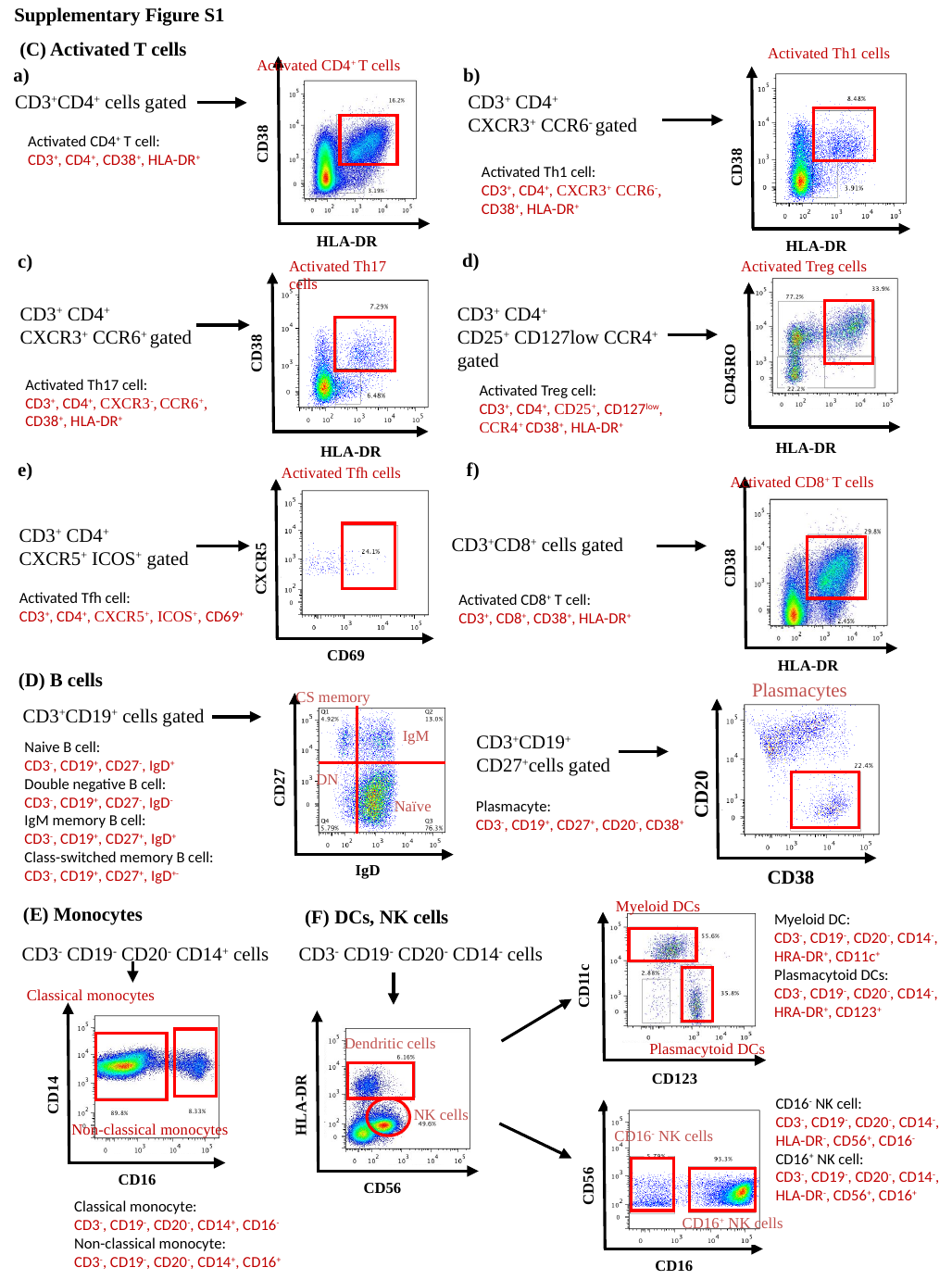

Supplementary Figure S1
(C) Activated T cells
Activated Th1 cells
CD38
HLA-DR
Activated CD4+ T cells
CD38
HLA-DR
b)
a)
CD3+ CD4+
CXCR3+ CCR6- gated
CD3+CD4+ cells gated
Activated CD4+ T cell:
CD3+, CD4+, CD38+, HLA-DR+
Activated Th1 cell:
CD3+, CD4+, CXCR3+ CCR6-,
CD38+, HLA-DR+
d)
c)
Activated Th17 cells
CD38
HLA-DR
Activated Treg cells
CD45RO
HLA-DR
CD3+ CD4+
CXCR3+ CCR6+ gated
CD3+ CD4+
CD25+ CD127low CCR4+
gated
Activated Th17 cell:
CD3+, CD4+, CXCR3-, CCR6+,
CD38+, HLA-DR+
Activated Treg cell:
CD3+, CD4+, CD25+, CD127low,
CCR4+ CD38+, HLA-DR+
e)
f)
Activated Tfh cells
CXCR5
CD69
Activated CD8+ T cells
CD38
HLA-DR
CD3+ CD4+
CXCR5+ ICOS+ gated
CD3+CD8+ cells gated
Activated Tfh cell:
CD3+, CD4+, CXCR5+, ICOS+, CD69+
Activated CD8+ T cell:
CD3+, CD8+, CD38+, HLA-DR+
(D) B cells
Plasmacytes
CD20
CD38
CS memory
IgM
DN
CD27
Naïve
IgD
CD3+CD19+ cells gated
CD3+CD19+
CD27+cells gated
Naive B cell:
CD3-, CD19+, CD27-, IgD+
Double negative B cell:
CD3-, CD19+, CD27-, IgD-
IgM memory B cell:
CD3-, CD19+, CD27+, IgD+
Class-switched memory B cell:
CD3-, CD19+, CD27+, IgD+-
Plasmacyte:
CD3-, CD19+, CD27+, CD20-, CD38+
 Myeloid DCs
CD11c
 Plasmacytoid DCs
CD123
(E) Monocytes
(F) DCs, NK cells
Myeloid DC:
CD3-, CD19-, CD20-, CD14-,
HRA-DR+, CD11c+
Plasmacytoid DCs:
CD3-, CD19-, CD20-, CD14-,
HRA-DR+, CD123+
CD3- CD19- CD20- CD14+ cells
CD3- CD19- CD20- CD14- cells
Classical monocytes
CD14
 Non-classical monocytes
CD16
Dendritic cells
HLA-DR
NK cells
CD56
CD16- NK cell:
CD3-, CD19-, CD20-, CD14-,
HLA-DR-, CD56+, CD16-
CD16+ NK cell:
CD3-, CD19-, CD20-, CD14-,
HLA-DR-, CD56+, CD16+
CD16- NK cells
CD56
CD16+ NK cells
CD16
Classical monocyte:
CD3-, CD19-, CD20-, CD14+, CD16-
Non-classical monocyte:
CD3-, CD19-, CD20-, CD14+, CD16+
